# Quality of nursing education programme in the Philippines: faculty members perspectives

**DOI:** 10.1186/s12912-020-00508-9

**Published:** 2020-11-25

**Authors:** Stella Appiah

**Affiliations:** School of Nursing and Midwifery, Valley View University, P. O. Box DT 595, Oyibi, Accra, Ghana

**Keywords:** Nursing education programme, Higher educational institutions, Faculty members

## Abstract

**Background:**

The subject of continuous improvement in the quality of nursing education programme is an extremely sensitive issue worldwide, particularly in the Philippines where a high number of trained registered nurses are exported to both developed and developing countries. The assessment of the quality of nursing education programme is usually measured using pass rates in licensure examinations by several government organizations. However, few studies have indicated that various categories of faculty members view the quality of nursing programmes differently, this study probed further and determined whether the quality of nursing education programme differs according to the profile of faculty members in Philippines colleges of higher education.

**Methods:**

A cross-sectional survey study design was employed in this study. One hundred and eight-five (185) faculty members in fifteen (15) higher educational institutions were selected for the research using purposive-census sampling. The study was carried out from January 1 to June 30, 2017. Close-ended structured questionnaires based on study objectives were used to collect data. Frequency and percentages were used to analyse the profile of faculty members whereas weighted means from a four Likert’s scale was used to interpret the extent of perceived quality of nursing education programme.

**Results:**

Majority, 39 and 46% of faculty members had 1–5 years’ clinical experience and 6–10 years of teaching experience respectively. Faculty members strongly agreed with a grand weighted mean of 3.84 out of 4.00 that nursing education programme is of good quality in the Philippines and is synonyms with other universities in the world. Teaching experience of faculty members showed significant relations in the quality of mission/vision/goals/objectives (*p*-value = 0.008), curriculum and instruction (*p*-value = 0.038), administration of nursing programme (*p*-value = 0.025), faculty development programme (*p*-value = 0.003), physical structure and equipment (*p*-value = 0.016), student services (*p*-value = 0.017), admission of students (*p*-value = 0.010) and quality assurance system (*p*-value = 0.009).

**Conclusion:**

Faculty members strongly perceived nursing education programme to be of good quality in this study. Teaching experience of instructors showed a significant relationship with the quality of nursing education programme in all the quality assessment indicators. However, clinical experience and job category of faculty members rather showed that the quality of nursing education programme is the same throughout all the higher educational institutions. The study implies that the teaching experience of faculty members is a strong predictor of quality of nursing education programme and employing faculty experienced in teaching is substantial for the continuous improvement of nursing education programme.

## Background

Quality of nursing education programme is a complex topic that involves the combination of teaching and learning materials, human resources, teaching and learning processes, curriculum, teaching and clinical experiences, teacher’s attitudes and commitment that are necessary to achieve set learning expectations and produce an exceptional performance of nursing students [1]. In essence, the nursing education programme can be classified as excellent or of high quality if it can be rated high (at least 80%) in all criteria used in its assessment.

The fundamental focus of nursing education programme is to produce nursing professionals that are clinically competent and can contribute immensely to the provision of quality and safe nurse care [2, 3]. Quality nursing education can be attained if nursing faculty obtain a balanced experienced in both theory and clinical areas. Consequently, this vast knowledge may results in nurses workforce that can apply the theory and lessons learnt in simulation laboratories into the delivery of health care in everyday living [4].

Worldwide, nurses are known to play a vital role in the rendering of health care services including many fundamental health-related services, particularly in rural areas. The increase of workforce without compromising on quality is imperative to help achieve Sustainable Development Goals (SDGs) targets that are set by the United Nations and its member countries, especially the one focused on universal health coverage [5, 6]. Quality of nursing education programme can be viewed as a long-term contributor to attaining quality universal health coverage in the production of qualified nurses.

The assessment of the quality of nursing education programme globally has usually been based on the pass rates in licensure examinations by several government organizations [7]. Although, several quality factors such as accreditation, students’ practical exposure and profile of faculty are sometimes considered to some extent [8]. The issue of quality of nursing education programme in Philippines colleges of higher education is extremely sensitive due to the high number of trained registered nurses exported to other countries such as the United States of America, United Arab Emirates and Germany [9, 10].

The quality of nursing education programme has been strongly associated with quality of curriculum, faculty and resources. These resources such as teaching materials and facilities are required to assist instructors in their delivery of nursing education [11]. The World Health Organization has also affirmed some standard criteria or area that quality of nursing education programme should be measured with of which faculty profile or development is included [12]. This activates the discussion into whether the quality of nursing education programme hinges on the profile of the faculty.

Aside from the challenge of quality of faculty members in nursing education programme, there are also problems of shortage of nursing instructors which usually affects the clinical teaching and learning environment and this clinical training feature of the nursing education programme results in long term detrimental effects in practising nurses if not well taught [13]. Also, other issues associated with quality of faculty members which consequently affects the quality of nursing education programme includes aging nursing faculty, less attractive faculty positions and length of education required to secure a faculty appointment [14].

According to a study conducted in Ghana, shortage of qualified instructors and insignificant upgrade of the previous infrastructure are critical issues facing the quality of nursing school and subsequently nursing education programme [15]. As far back as 27 years ago, some authors have connected the quality of nursing education in the aspect of teaching and practical gap to nursing faculty inability to assume a commanding role in clinical learning and teaching [16]. Earlier researches have related quality of nursing education to the quality of nursing instructors and this study, therefore, determined the quality of nursing education programme. This paper also found out whether the quality of nursing education programme differs according to the profile of faculty members using Philippines colleges of higher education as a case study, in addressing this, the study was based on the null hypothesis (Ho), there is no significant difference in the quality of nursing education programme by the respondents (faculty members) when grouped according to their profile.

## Methods

### Study design

A cross-sectional survey study design was used in this research. This was executed by employing a purposive-census sampling method to recruit one hundred and eighty-five (185) faculty instructors in fifteen (15) higher educational institutions. The use of Purposive sampling was based on the belief that the researcher’s knowledge about the population can be used to hand-pick respondents [17] and in this case HEIs. This approach did not necessarily mean HEIs known to the researcher were selected but those who met the criteria of inclusion were used for the study. Census sampling, on the other hand, is the process of taking the total population of the locale and retrieving an adequate number of respondents (faculty) as a sample of the study. The use of the two approaches complimented each other for the recruitment of the HEIs and study respondents Questionnaires centred on the aim and study objectives of the study were self-administered after the consent of participants was sought. The study was carried out with the period of January 1 – June 30, 2017.

### Study area

The study was conducted in fifteen (15) private-owned higher educational institutions in the National Capital Region (NCR) of the Philippines. Ten out of fifteen of these higher educational institutions had existed for more than 45 years whilst the rest had been in existence for less than 45 years. Although 7 of these institutions were granted autonomous by a regulating body called CHED, 8 of them were still monitored by the same regulatory agency. Also, 12 of these institutions were owned by private non-sectarian organizations, however, 3 were owned by private sectarian establishments. The central government seat is in the National Capital Region and the city holds the highest number of higher education institutions which comprises those offering nursing education programme. Majority of these institutions offer health-related programmes at both undergraduate and postgraduate levels, however, the study concentrated on the nursing education programme at the undergraduate level.

### Sampling procedure

An initial number of all twenty-two (22) recognized higher national institutions owned by private entities in the NCR were contacted to take part in the study, yet, a considerable number of fifteen (15) institutions approved for their school and faculty members to partake in the study. The number of faculty in the twenty-two (22) higher national institutions targeted for the study summed up to two hundred and twenty (220), nonetheless, one hundred and eighty-five (185) faculty members consisting of deans, program coordinators, and faculty instructors on full and part-time contracts consented and were recruited into the study. The made the study achieve a response rate of 84.1%.

### Inclusion criteria

All teaching and clinical instructors who have spent more than 1 year in their educational institution were recruited to partake in the study.

### Exclusion criteria

All other instructors who had not completed 12 months in their educational institutions were not allowed to be part of the study.

### Tool for data collection

A questionnaire was specifically developed to undertake this study. In doing so, three areas were considered in the design of a close-ended questionnaire used for the study. These were; 1) aim and objectives of study 2) policies and standards of nursing schools in the Philippines and 3) World Health Organization (WHO) guidelines on quality assurance and accreditation of nursing and midwifery educational institutions in the South-East Asian countries. A four (4) Likert scale with standard questions were used to evaluate the quality of nursing education programme. The criteria for the assessment of the quality of nursing education programme included mission/vision/goals/objectives, curriculum and instruction, administration of nursing education, faculty development programme, physical structure and equipment, student services, admission of students and quality assurance system. Pretesting of the questionnaire was done in one of the accredited colleges of nursing in NCR with nineteen (19) respondents to measure the reliability of the tool before it was employed for the study. In doing so, the questionnaire was subjected to Cronbach’s alpha reliability test to determine its consistency and validity. The overall result showed .989 indicating a high consistency and reliability.

### Data analysis

Information from the completed questionnaire was entered into Microsoft Excel and imported into SPSS statistical software version 22 for editing, cleaning and analysis. Frequency and percentage were employed to analyse the profile of faculty members while weighted means from a four-Likert scale was used to interpret the extent of perceived quality of nursing education programme as assessed by faculty members. The scales for assessing the quality of nursing education programme; 1.00–1.49, 1.50–2.49, 2.50–3.49 and 3.50–4.00 was interpreted as strongly disagree, disagree, agree and strongly agree respectively. One-way ANOVA was used to test for the differences in quality of nursing education programme in the eight (8) thematic areas (mission/vision/goals/objectives, curriculum and instruction, administration of nursing education, faculty development programme, physical structure and equipment, student services, admission of students and quality assurance system) concerning the profile of faculty members. A *P*-value of less than 0.05 was considered significant in this study.

## Results

### Profile of faculty members in higher educational institutions (HEIs)

A majority, 73 (39.0%) of the 185 participants that partook in the study had 1–5 years’ clinical experience whilst few, 15 (8.0%) had 16–20 years of clinical experience. Almost half, 85 (46.0%) of respondents had taught for 6–10 years nonetheless a small number, 14 (8.0%) had 16–20 years of teaching experience. A little below two-thirds, 121 (65.0%) were doing both clinical and classroom teaching whereas very few, 8 (4.0%) were deans of the nursing department in their institutions (Table 1).

**Table 1:** Profile of faculty members in higher educational institutions (HEIs)

Profile of Respondents	Frequency (185)	Percentage (%)
Years of Clinical Experience		
1–5	73	39.0
6–10	47	25.0
11–15	22	12.0
16–20	15	8.0
≥ 21	28	15.0
**Years of Teaching Experience**		
≤ 5	16	9.0
6–10	85	46.0
11–15	46	25.0
16–20	14	8.0
≥ 21	24	13.0
**Job Category**		
Classroom faculty	12	6.0
Classroom-clinical	121	65.0
Clinical instructor	22	12.0
Program Coordinator	22	12.0
Dean	8	4.0

### Quality of nursing education Programme as perceived by faculty members

At the end of the assessment of the nursing education programme, an average of the grand weighted mean of 3.84 resulted, which means participants strongly agreed that nursing programme is of good quality. However, administrators who were faculty members rated the quality of nursing education programme higher with a mean of 3.88 compared to a mean of 3.81 by faculty who were only instructors. The quality of the mission/vision/goals/objectives of the nursing education programme was appraised highest with a mean of 3.91 while the least valued was the admission of students with a mean of 3.76 (Table 2).

**Table 2:** Quality of nursing education as perceived by faculty members

Quality Matrix	Faculty		Administrator		Average	
	WM	QD	WM	QD	WM	QD
Mission/Vision/Goals/Objectives	3.89	SA	3.93	SA	3.91	SA
Curriculum and instruction	3.86	SA	3.94	SA	3.90	SA
Administration of nursing programme	3.81	SA	3.94	SA	3.88	SA
Faculty development program	3.84	SA	3.88	SA	3.86	SA
Physical structure and equipment	3.81	SA	3.80	SA	3.81	SA
Student services	3.81	SA	3.96	SA	3.88	SA
Admission of students	3.73	SA	3.78	SA	3.76	SA
Quality assurance system	3.72	SA	3.84	SA	3.78	SA
**Grand Weighted Mean (WM)**	3.81	SA	3.88	SA	3.84	SA

Legend: 1.00–1.49 Strongly Disagree (SD), 1.50–2.49 Disagree (D), 2.50–3.49 Agree (A), 3.50–4.00 Strongly Agree (SA), Grand Weighted Mean (WM), Qualitative Description (QD)

### The difference in quality of nursing education Programme according to profile of faculty

Significant differences existed in the quality of mission/vision/goals/objectives (*p*-value = 0.008), curriculum and instruction (*p*-value = 0.038), administration of nursing programme (*p*-value = 0.025), faculty development programme (*p*-value = 0.003), physical structure and equipment (*p*-value = 0.016), student services (*p*-value = 0.017), admission of students (*p*-value = 0.010) and quality assurance system (*p*-value = 0.009) in relation to teaching experience of faculty members. On the contrary, the quality of mission/vision/goals/objectives, curriculum and instruction, administration of nursing education, faculty development programme, physical structure and equipment, student services, admission of students and quality assurance system did not differ significantly with regards to clinical experience and job category of faculty instructors (Table 3).

**Table 3:** Difference in Quality of Nursing Educational Program according to Profile of Faculty

Profile of HEIs	Quality Matrix	F	*p*-value	Decision (Ho)	Interpretation
Clinical experience	Mission/Vision/Goals/Objectives	2.332	0.058	Accept	Not significant
	Curriculum and instruction	0.862	0.488	Accept	Not significant
	Administration of nursing programme	0.672	0.612	Accept	Not significant
	Faculty development program	0.666	0.617	Accept	Not significant
	Physical structure and equipment	0.705	0.589	Accept	Not significant
	Student services	0.763	0.828	Accept	Not significant
	Admission of students	0.302	0.876	Accept	Not significant
	Quality assurance system	0.694	0.597	Accept	Not significant
Teaching experience	Mission/Vision/Goals/Objectives	3.600	0.008*	Reject	Significant
	Curriculum and instruction	2.592	0.038*	Reject	Significant
	Administration of nursing programme	2.856	0.025*	Reject	Significant
	Faculty development program	4.162	0.003*	Reject	Significant
	Physical structure and equipment	3.128	0.016*	Reject	Significant
	Student services	3.104	0.017*	Reject	Significant
	Admission of students	3.421	0.010*	Reject	Significant
	Quality assurance system	3.471	0.009*	Reject	Significant
Job category	Mission/Vision/Goals/Objectives	1.046	0.376	Accept	Not significant
	Curriculum and instruction	1.107	0.355	Accept	Not significant
	Administration of nursing programme	1.734	0.145	Accept	Not significant
	Faculty development program	1.171	0.325	Accept	Not significant
	Physical structure and equipment	1.114	0.219	Accept	Not significant
	Student services	1.891	0.114	Accept	Not significant
	Admission of students	1.453	0.218	Accept	Not significant
	Quality assurance system	0.538	0.708	Accept	Not significant

The *p*-values denoted by ‘*’ are significant at a level of *p* < 0.05, Ho – denotes null hypothesis

## Discussion

The excellence of nursing education programme and to a large extent nursing institution has often been linked to success in licensure exams undertaken by nursing students while other studies have associated it to the quality and shortage of nursing instructors [7, 13]. The findings of this study also sought to unravel another concept of whether the profile of faculty in terms of clinical experience, teaching experience and job category cause significant differences in the quality of nursing education programme in the areas of mission/vision/goals/objectives, curriculum and instruction, administration of nursing education, faculty development programme, physical structure and equipment, student services, admission of students and quality assurance system.

According to this study, participants strongly agreed with a score of 3.84 out of 4.00 that quality of nursing education programme offered by institutions in the Philippines is similar to ones run by other universities. This high grading of the quality of nursing education programme may have resulted because of the majority, 39 and 46% of faculty members had 1–5 years’ clinical experience and 6–10 years of teaching experience respectively. Also, this is consistent with the requirement that nursing instructors should have at least a year each of clinical and teaching experience [18, 19]. Again, clinical experience particularly is important to close the gap between classroom lessons and simulation classes and in the long run improves the quality of nursing education programme [4].

Besides, this study also found that about two-thirds, 65% of faculty members were both classroom and clinical instructors. This result has a direct influence on the high grading of the quality of nursing programme because is very suitable if instructors who taught a particular group of students takes them through the practical component of the course. This leads to a better delivery of the curriculum of nursing education programme according to [11], which consequently leads to a high quality of nursing education programme.

The World Health Organization emphasized vision as a key requirement for the quality of nursing education as part of the global standards for the education of professional nurses and midwives [12]. In a study on quality assurance in higher education, mission/vision/goals/objectives were rated higher as the driving force for the quality nursing education programme. This was also congruent with the finding of this study where mission/vision/goals/objective was rated highest, 3.91 out of 4.00 compared to the other quality matrix that was used in the assessment of nursing education programme.

Even though, many studies have emphasized on the importance of clinical experience on the quality of nursing education programme [4], clinical experience and type of faculty did not show a significant difference in the quality of nursing education programme in all the eight quality matrix in this study. This implies that the quality of nursing education programme will be the same throughout all the nursing colleges in the Philippines with regards to clinical experience and job category of instructors.

Conversely, teaching experience of faculty revealed a significant difference in the quality of nursing education programme in all the criteria used for monitoring the quality of nursing education programme. This underlines the importance of teaching experience in the quality of nursing education [20]. Experienced faculty members are usually needed in the development of mission/vision/goals/objectives, curriculum and instruction of a nursing programme [21]. They are also practically involved in the administration of nursing education programme and even report gaps in a curriculum where and when is necessary [21, 22]. The nursing lecturers who have enough teaching experience had usually undergone series of faculty development programme and have the experience in advising management on the needed physical structure and equipment that are necessary for the running of quality nursing education programme [23]. Also, these faculty members have the knowledge in the calibre of students to be admitted and the type of student services that should be provided to ensure the best of nursing education. This finding on the teaching experience of faculty members discloses its importance on the quality of nursing education programme and hence worth considering in enlisting processes.

## Conclusion

Faculty members strongly perceived nursing education programme to be of good quality in this study. Majority of these nursing instructors had sufficient years of both clinical and teaching experience. No significant difference was found in all the quality criteria of nursing education programme with regards to the profile of instructors; clinical experience and job category. However, teaching experience revealed a significant difference in the quality of nursing education programme in the area of mission/vision/goals/objectives, curriculum and instruction, administration of nursing education, faculty development programme, physical structure and equipment, student services, admission of students and quality assurance system.

## Recommendation

The study, therefore, encourages management of higher educational institutions to emphasize teaching experience as one of the criteria that merit consideration for the recruitment of faculty members for a nursing education programme. This will guarantee continuous improvement of quality of nursing education programme in higher educational institutions in the Philippines and other countries.

### Limitation

Although Likert scale was used by study participants to measure quality of nursing education programme in all the criteria in the quality matrix used in this study, respondents may have been biased in the answering of questions concerning nursing education programme using this scale where they intentionally avoid extreme answers and choose options that are expected than the real situation the study sought to find.

## Supplementary Information

**Additional file 1.** Quality of nursing education programme assessment tool. A four Likert scale questionnaire for the assessment of nursing education programme on eight thematic areas.

## Data Availability

The study data and materials are in the custody of the corresponding author and can be made available on reasonable request.
