# Supplementary material for: Quality of nursing education programme in the Philippines: faculty members perspectives
Source: BMC Nurs. 2020 Nov 25;19:110. doi: 10.1186/s12912-020-00508-9 (PMC7689987; doi:10.1186/s12912-020-00508-9)
Supplement: Supplementary file 1 — Additional file 1. Quality of nursing education programme assessment tool. A four Likert scale questionnaire for the assessment of nursing education programme on eight thematic areas. [file 12912_2020_508_MOESM1_ESM.docx]

**Quality of nursing education programme assessment tool**

| **Quality matrix** | **4** | **3** | **2** | **1** |
| --- | --- | --- | --- | --- |
| **Mission/Vision/Goals/Objectives of the program** | | | | |
| 1. Mission, vision, goals, objectives and Philosophy of the nursing program is congruent with those of the University. |  |  |  |  |
| 1. The mission, vision, goals and objectives of the nursing program is clearly stated. |  |  |  |  |
| 1. The administrative and academic staff and students understand the mission, vision, goals and objectives of the nursing program. |  |  |  |  |
| 1. Faculty, administrators and students participate in governance as defined by nursing program. |  |  |  |  |
| 1. Nursing program is administered by a nurse who is academically qualified and has experience. |  |  |  |  |
| 1. The authority and responsibility of the nursing program is administered by a qualified nurse administrator. |  |  |  |  |
| 1. Policies of the nursing program are consistent with those of the Commission on Higher Education (CHED). |  |  |  |  |
| **Curriculum and instruction of the Program** | **4** | **3** | **2** | **1** |
| 1. Curriculum developed flows from the philosophy/mission, vision, goals and objectives of the nursing program through an organizational framework into a logical progression of course outcomes and learning activities to achieve desired program objectives/outcomes. |  |  |  |  |
| 1. The nursing program conforms to the CHED standards of nursing curriculum. |  |  |  |  |
| 1. The program designed provides opportunities for students to achieve program objectives, skills, values and competencies necessary for the practice of nursing. |  |  |  |  |
| 1. The faculty use appropriate and updated course syllabi, instructional or teaching methodology. |  |  |  |  |
| 1. Practice learning environments are selected and monitored by the HEI. |  |  |  |  |
| 1. There is a provision for opportunities of exposure in a variety of related learning experiences appropriate for contemporary nursing. |  |  |  |  |
| 1. The nursing program maintains high level of instruction. |  |  |  |  |
| 1. The teaching learning process is composed of theoretical and experiential. |  |  |  |  |
| **Administration of the nursing program** | **4** | **3** | **2** | **1** |
| 1. The composition, responsibilities and function of the general administrative body are defined in writing. |  |  |  |  |
| 1. The nursing program is administered by a qualified administrator as specified in the CHED CMO. |  |  |  |  |
| 1. The nursing program administrator is a registered nurse with leadership competencies. |  |  |  |  |
| 1. The level coordinator, program coordinator and deans are top-level decision makers and are leaders of the nursing program. |  |  |  |  |
| 1. The general administrative body holds regular meetings. |  |  |  |  |
| 1. Minutes of administrative meetings and pertinent information are communicated to staff. |  |  |  |  |
| 1. The organization is in compliance with all applicable legislation, including the provisions of CHED. |  |  |  |  |
| 1. The organizational structure is outlined in an organizational chart. |  |  |  |  |
| 1. The organizational structure and organizational chart is regularly reviewed and recorded. |  |  |  |  |
| **Faculty development program** | **4** | **3** | **2** | **1** |
| 1. Nursing program faculty members (fulltime and part-time) are academically qualified and have experience. |  |  |  |  |
| 1. Nursing program faculty members maintain expertise in their areas of responsibility and teaching skills. |  |  |  |  |
| 1. Adequate number of full-time and part-time staff meets the needs of the nursing program to ensure competency. |  |  |  |  |
| 1. Faculty performance is periodically evaluated to ensure ongoing development and competence. |  |  |  |  |
| 1. The faculty who teach nursing core courses has an educational background in nursing. |  |  |  |  |
| 1. Teaching load is appropriate. (Suggested load is 36 hours per week for lectures and Related Learning Experience (RLE) for full time faculty and 9 hours for part time faculty). |  |  |  |  |
| 1. The collective talents of the nursing program faculty reflect scholarship through teaching, application, and the integration and discovery of knowledge as defined by the institution and the nursing program. |  |  |  |  |
| 1. Faculty is evaluated on their performance regularly. |  |  |  |  |
| 1. The HEI has a five-year faculty development plan. |  |  |  |  |
| **Physical structure and equipment** | **4** | **3** | **2** | **1** |
| 1. Classroom for regular lecture contains maximum of 50 students. |  |  |  |  |
| 1. Science laboratory class size has a maximum of 25 students. |  |  |  |  |
| 1. Special classes can accommodate up to 45 students when made available by the facilities. |  |  |  |  |
| 1. Nursing laboratory room is well-ventilated and lighted for students to have enough space for practice and return demonstration. |  |  |  |  |
| 1. The laboratory is equipped with basic instruments for learning purposes. |  |  |  |  |
| 1. Physical facilities including information technology (IT) and environment are safe, clean and appropriate to support the purposes of the nursing program. |  |  |  |  |
| 1. Clinical resources including hospital and community personnel, patient–student ratio are appropriate. |  |  |  |  |
| 1. Nursing equipment are adequate to support the purpose of the nursing program |  |  |  |  |
| 1. Students are provided opportunities to practice in a variety of essential areas. |  |  |  |  |
| 1. The library has adequate and up to date textbooks for faculty and students use. (Suggested 5 copies per title and not more than 5 years old). |  |  |  |  |
| 1. The library has journals and other resources for faculty and students use. |  |  |  |  |
| **Student services** | **4** | **3** | **2** | **1** |
| 1. Student policies of the nursing program are congruent with those of the university. |  |  |  |  |
| 1. Student policies of the nursing program are publicly accessible, non-discriminatory and consistently applied. |  |  |  |  |
| 1. Students have access to support services provided by qualified individuals which include, but are not limited to: Health, counseling, academic advancement, career, library, placement and financial aid. |  |  |  |  |
| 1. Policies concerned with educational and financial records are established and followed |  |  |  |  |
| 1. Plans for student activity and development are indicated. |  |  |  |  |
| 1. Students are supported to develop a student club and engage in extracurricular activities. |  |  |  |  |
| 1. The academic adviser arranges time for students to meet her/him and advise them at least once a term. |  |  |  |  |
| 1. There is availability of internal, external rewards/scholarships for excellent students and Dean’s list of excellence. |  |  |  |  |
| **Admission of students** | **4** | **3** | **2** | **1** |
| 1. Admission criteria and policy are clearly stated. |  |  |  |  |
| 1. Recruitment methods of student well indicated |  |  |  |  |
| 1. Number of students enrolled is adequate. |  |  |  |  |
| 1. Students are recruited based on a written test |  |  |  |  |
| 1. Qualified students are not discriminated against on the bases of colour, race, religion etc. |  |  |  |  |
| **Quality assurance system** | **4** | **3** | **2** | **1** |
| 1. There is a written plan for a systematic quality assurance programme for nursing program, which is used for continuous programme improvement. |  |  |  |  |
| 1. There is a written plan for systematic evaluation of the program’s purposes and product outcomes |  |  |  |  |
| 1. There is an appointed QA committee personnel unit, and internal auditing and monitoring control |  |  |  |  |
| 1. There is documentation of the implementation plan of QAS. |  |  |  |  |
| 1. There are available methods for monitoring and evaluating the programme. |  |  |  |  |
